# Chronic physical exercise alleviates stress-associated amygdala metabolic activity in obese women: A prospective serial ^18^F-FDG PET/CT study

**DOI:** 10.3389/fendo.2022.1046838

**Published:** 2023-01-05

**Authors:** Kisoo Pahk, Chanmin Joung, Hyun Woo Kwon, Sungeun Kim

**Affiliations:** ^1^ Department of Nuclear Medicine, Korea University Ansan Hospital, Ansan, South Korea; ^2^ Department of Nuclear Medicine, Korea University Anam Hospital, Seoul, South Korea; ^3^ Graduate School of Biomedical Sciences, University of Texas Southwestern Medical Center, Dallas, TX, United States

**Keywords:** obesity, cardiovascular disease, amygdala, stress, exercise, positron-emission tomography

## Abstract

**Background:**

Psychological stress is considered as a major risk factor for cardiovascular disease (CVD). Chronic exercise is known to reduce CVD risk partly through attenuating psychological stress. Obesity has been linked with increased levels of psychological stress. We aimed to prospectively evaluate whether physical exercise could alleviate stress-associated amygdala metabolic activity, assessed by ^18^F-fluorodeoxyglucose (FDG) positron emission tomography/computed tomography (PET/CT) in women with obesity.

**Material and methods:**

A total of 43 participants were enrolled in this study. Twenty-three obese women were participated in a physical exercise program 5 days per week for 3 months. The exercise program consisted of aerobic exercise and resistance training. Serial ^18^F-FDG PET/CT was taken before the start of physical exercise program (baseline) and after finishing the program (post-exercise). A total of 20 participants who underwent ^18^F-FDG PET/CT for general health check-up were enrolled as non-obese control group. Brain amygdala activity (AmygA) was calculated as maximum standardized uptake value (SUVmax) of amygdala normalized to mean SUV of temporal lobe.

**Results:**

Chronic physical exercise significantly reduced AmygA and improved body adiposity and systemic inflammation. AmygA was highest in baseline, intermediate in post-exercise, and lowest in non-obese control group (0.76 ± 0.17, 0.61 ± 0.1, 0.52 ± 0.09, *p* < 0.001). Furthermore, physical exercise also abrogated the association of AmygA with systemic inflammation.

**Conclusions:**

Chronic physical exercise reduced stress-associated amygdala metabolic activity and broke its association with systemic inflammation in obese women. This study could explain the putative mechanism underlying the health beneficial effect of exercise on CVD *via* attenuation of stress neurobiology.

## Introduction

Psychological stress, an essential component of life in human beings, is the physiologic emotional response to physiological-, social-, and environmental stimuli stressors (1). In recent years, psychological stress has been increasingly considered as an independent risk factor for cardiovascular disease (CVD), which is the leading cause of global death (2, 3). Furthermore, psychological stress is also associated with well-known traditional CVD risk factors such as obesity and hypertension (4, 5). Psychological stress has been considered as one key factor in the development of obesity and people with obesity show elevated levels of psychological stress (4, 5). However, detailed underlying mechanism that translate psychological stress into CVD risks remains to be fully explored.

Recently, efforts to elucidate the underlying mechanism of stress and CVD risk have focused on specific brain region which may activated by psychological stress (6). Amygdala, a core part of brain’s salience network, is known to regulate pathophysiologic and behavioral response to psychological stress with accompanied increased in pro-inflammatory cytokine levels (7, 8). Accumulating evidence have suggested that resting amygdala activity (AmygA) can be reproducibly measured with high reliability using ^18^F-fluorodeoxyglucose (FDG) positron emission tomography/computed tomography (PET/CT) (9–11). AmygA has been well correlated with stress-related psychometric questionnaires (10, 11) and was associated with greater circulating systemic inflammatory surrogate markers and with worsening atherosclerotic plaque vulnerability (10). Furthermore, AmygA independently predicts subsequent CVD events (9, 12). Thus collectively, AmygA can reflect the risk burden of CVD imposed by psychological stress.

Chronic physical exercise is well-known to exert substantial health benefits and attenuates CVD risk, partly through its anti-inflammatory activities on brain, adipose tissue, and hematopoietic organs (13). In addition, chronic exercise also reduces the level of stress, as assessed by psychometric questionnaires, and attenuates systemic inflammation (14). However, little is known about the effect of chronic physical exercise on stress-associated neurobiological activity. As AmygA has been associated with systemic inflammation, which is mitigated by exercise, we hypothesized that AmygA could also be affected by chronic physical exercise.

In present study, we aimed to investigate whether chronic physical exercise could alleviate AmygA, evaluated by ^18^F-FDG PET/CT in obese women and could affect the relationship between AmygA and systemic inflammation.

## Materials and methods

### Study design and population

**Figure 1:**
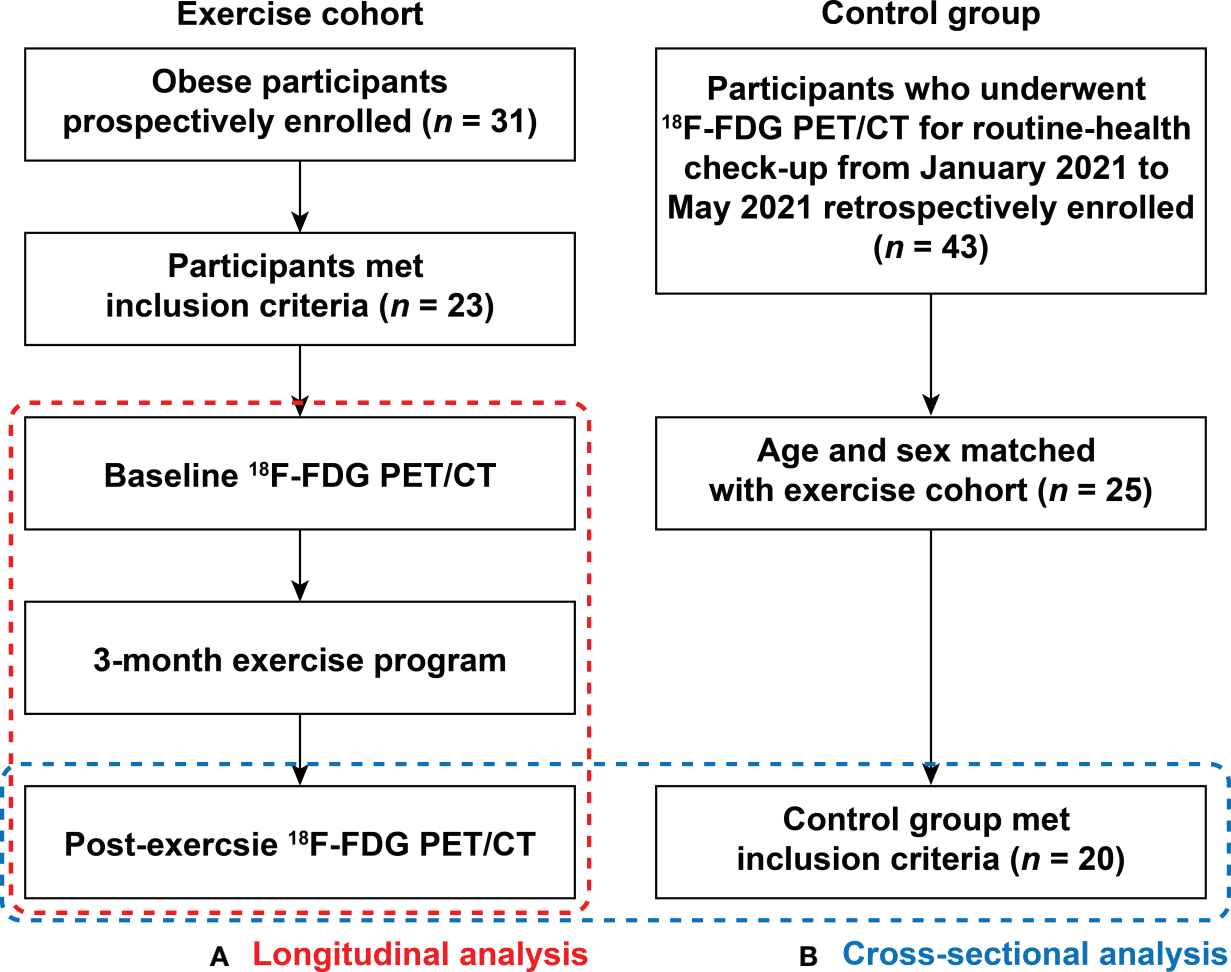
Flow chart showing enrollment scheme. This study was composed of two types of investigations: **(A)** Longitudinal analysis within the obese women exercise cohort from baseline to 3-month after completion of scheduled exercise; and **(B)** Cross-sectional analysis of post-exercise obese women and non-obese control group. ^18^F-FDG PET/CT, ^18^F-fluorodeoxyglucose (FDG) positron emission tomography/computed tomography (PET/CT).

This study was composed of two types of investigations. As shown in **Figure 1**, the first was a longitudinal study exploring the temporal change of AmygA after chronic physical exercise in obese women. The second was a cross-sectional study comparing AmygA between obese women who completed the chronic physical exercise program and non-obese control group.

The longitudinal study population was recruited between June 2008 to March 2009 from the local community health center. Obesity was defined as a body mass index (BMI) of at least 25 kg/m^2^, according to the guideline for the management of obesity in Korea (15). Participants with a history of malignancy, those who had previously diagnosed CVD, hypertension (≥ stage 2), uncontrolled diabetes mellitus, autoimmune disease, severe hepatic, or renal disease, and those had taken any medications that could affect systemic inflammation within 6 months were excluded. Finally, a total of 23 participants were enrolled in this longitudinal study. They all underwent scheduled exercise program under supervision (5 times a week for 3 months) and without restricting diet. ^18^F-FDG PET/CT was performed before the start of exercise program (baseline) and after completion of 3 months of exercise program (post-exercise). In cross-sectional study, age-and sex matched non-obese control participants who underwent ^18^F-FDG PET/CT for general health check-up were enrolled from the Health Promotion Center at our hospital between January 2021 and May 2021. The exclusion criteria were same as the above longitudinal study. In total, 20 women were enrolled as non-obese control group. This study was approved by the Ethical Committee and Institutional Review Board of Korea University Hospital (Approval No. GR0888-005) and all participants provided written informed consent.

### Exercise protocol

The exercise program was designed to include aerobic- with subsequent muscle-resistant exercise, recommend by the American Heart Association (AHA) and the American College of Sports Medicine (ACSM) to improve and keep health (16). For aerobic exercise, participants engaged in 30 min of moderate-intensity activity (walking at very brisk pace at 4 mph) followed by 20 mins of vigorous-intensity activity (running at 7 mph). Using metabolic equivalents (METs), intensity between 3 and 6 METs was considered moderate-intensity and over 6 METs was considered vigorous-intensity, respectively (16). For muscle-resistant exercise, participants engaged in 8-10 exercises involving major muscle groups with 8 to 12 repetitions, as previously described (16).

### Anthropometric and laboratory measurements

BMI was determined as weight/height squared (kg/m^2^). Waist circumference was measured at the narrowest point between the last rib and the top of iliac crest after normal exhaling. Hip circumference was measured at the maximum protrusion of buttocks. Blood samples were obtained after 12-h overnight fasting, and analyzed with chemistry analyzer (Hitachi 747, Hitachi, Tokyo, Japan). The levels of high-sensitivity C-reactive protein (hsCRP) were analyzed with Dade Behring BNII analyzer (Siemens, Munich, Germany).

### ^18^F-FDG PET/CT imaging acquisition protocol

^18^F-FDG was injected intravenously at a dose of 5.29 MBq/kg after an overnight fast for at least 8 h. PET/CT images were acquired 1 h after tracer injection with an integrated scanner (GEMINI TF, Philips Medical Systems, Cleveland, OH, USA). Non-contrast-enhanced CT (120 kVp, 50mA, 4 mm thickness) was performed for attenuation correction before PET scan. The scanning range was set from vertex of skull to proximal thigh. All PET images were reconstructed by 3D-ordered iterative algorithm (3 iterations with 33 subsets, matrix size 144 × 144).

### Image analysis

Images were analyzed by two fully trained nuclear medicine radiologists (KP and HWK), who were blinded to clinical data, using a commercially available workstation (Extended Brilliance Workspace version 3.5, Philips Healthcare, Eindhoven, Netherlands). ^18^F-FDG uptakes were quantified by using standardized uptake value (SUV) which is defined as the tissue radioactivity concentration (MBq/g) in a region of interest (ROI) normalized for the injected dose (MBq) and the total body weight (g).

The amygdalae, which are the part of brain’s limbic system deep within the temporal lobe, forming the ventral, superior and medial aspect of the inferior horn of the lateral ventricle, were recognized by anatomical landmarks which were described in previous studies (9–12). In detail, the anterior and the posterior boundaries were identified as the inferior aspect of the lateral ventricles and the crus of fornix forming anterior to the basilar artery, respectively. Both lateral and inferior boundary were determined by the internal capsule. Next, for the evaluation of AmygA, circular ROIs were placed on both left and right amygdala and SUVs were measured, as previously described (9–12). AmygA was defined by dividing the maximum SUVs (SUVmax) in each amygdala by the mean SUVs in ipsilateral brain temporal lobes for normalization of background activity. The primary measure of AmygA was the highest AmygA between the two amygdalae (9–12).

Metabolic activities in hematopoietic system such as spleen and bone marrow (BM) are the well-known surrogate markers to reflect the systemic inflammation (17–19). ROIs were placed within spleen and vertebrae (L3 to L5) to measure spleen and BM metabolic activity. Spleen SUVmax was defined as the average SUVmax of spleen on all axial slices. BM SUVmax was defined as the average SUVmax of L3 to L5 vertebrae (18, 19).

### Statistical analysis

Data are presented as mean and standard deviation. Shapiro-wilk test was used to determine the normalcy of variables. Paired *t*-test or Wilcoxon signed-rank test was used for comparison between baseline and post-exercise. For comparison of two groups, Student’s *t*-test, or Mann–Whitney *U* test was used. The Pearson Chi squared (χ2) test or Fisher’s exact test, Spearman’s correlation analysis, univariate-, and multivariate linear regression were also performed as statistical methods. MedCalc software version 18.5 (MedCalc Software Ltd, Ostend, Belgium) and SPSS software version 17.0 (SPSS Inc, Chicago, IL, USA) were used for data analysis. A *p*-value equal or less than 0.05 was considered statistically significant.

## Results

**Figure 2:**
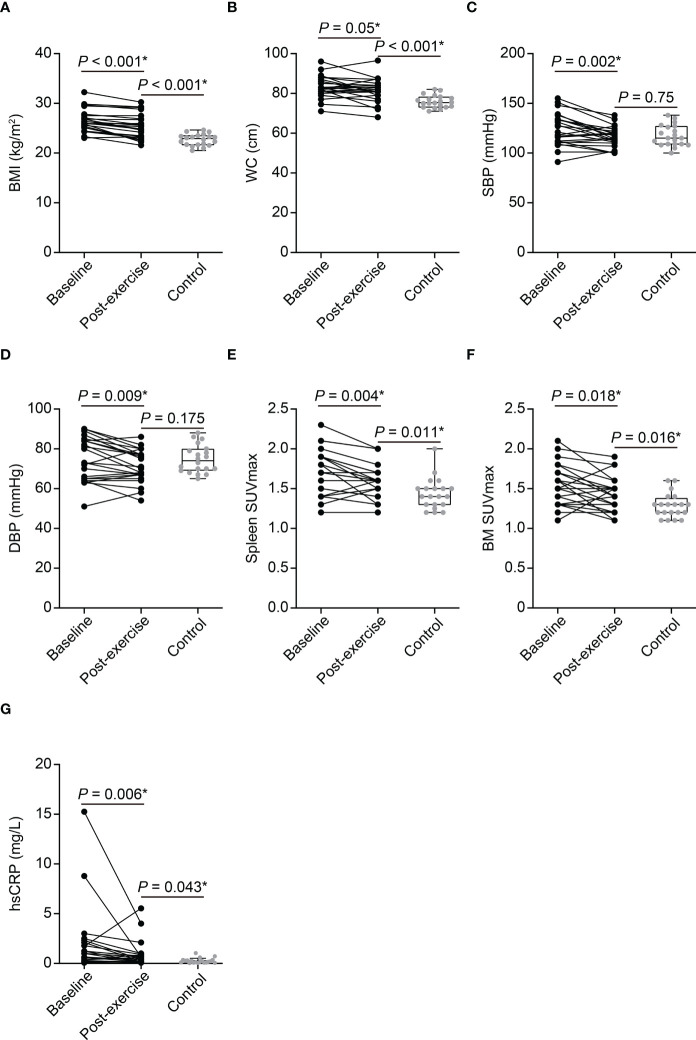
The effect of exercise on clinical parameters of obese women and comparison between post-exercise obese women and control participants **(A–G)**. BMI, body mass index; WC, waist circumference; SBP, systolic blood pressure; DBP, diastolic blood pressure; SUVmax, maximum standardized uptake value; BM, bone marrow; hsCRP, high-sensitivity C-reactive protein. *P*-values of BMI, WC, SBP, DBP, spleen SUVmax, BM SUVmax, and hsCRP between baseline and post-exercise obese women were determined using paired *t*-test. *P*-values of BMI, WC, SBP, DBP, spleen SUVmax, and BM SUVmax between post-exercise obese women and control group were determined using Student’s *t*-test. *P*-value of hsCRP between post-exercise obese women and control group was determined using Mann–Whitney *U* test. *Statistically significant difference.

All enrolled obese women have completed the scheduled exercise program and detailed baseline characteristics of the study population are presented in **Table 1**. As shown in **Figure 2**, a 3-month of chronic exercise significantly alleviated blood pressure and body adiposity.

**Table 1:** Baseline characteristics of obese women.

Baseline characteristics	n = 23
Age (years)	46 ± 8.0
Height (cm)	156.8 ± 5.5
Alcohol drinking, n (%)	8 (34.8)
Smoking (current), n (%)	0 (0)
Medication, n (%)	0 (0)
Diabetes, n (%)	0 (0)
Dyslipidemia, n (%)	7 (30.4)
Hypertension (stage I), n (%)	4 (17.4)
Menopause, n (%)	12 (52.2)

Age and height were expressed as mean ± standard deviation.

### Chronic physical exercise reduces AmygA and systemic inflammation in obese women

**Figure 3:**
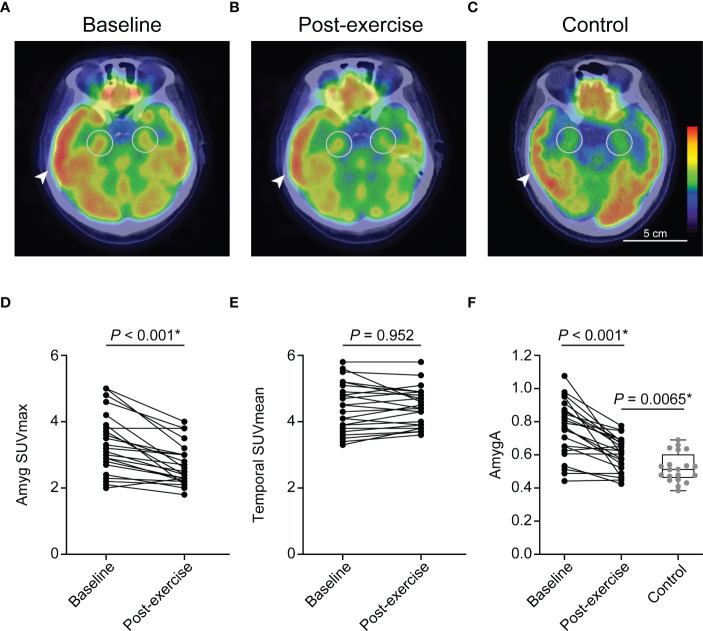
Chronic physical exercise significantly reduced the amygdala activity (AmygA) in obese women. Representative fused images of ^18^F-FDG PET/CT at baseline- **(A)**, post-exercise obese women **(B)**, and non-obese control group **(C)**. Changes in Amyg SUVmax **(D)** and Temporal SUVmean **(E)** between baseline and post-exercise obese women. Changes in AmygA between baseline and post-exercise obese women, and comparison of AmygA between post-exercise obese women and non-obese control group **(F)**. Amyg SUVmax, maximum standardized uptake value of amygdala; Temporal SUVmean, mean standardized uptake value of temporal lobe; AmygA, Amyg SUVmax/Temporal SUVmean. The circular ROI indicates amygdala, and the arrowhead indicates temporal lobe. *P*-values of Amyg SUVmax, Temporal SUVmean, and AmygA between baseline and post-exercise obese women were determined using paired *t*-test. *P*-value of AmygA between post-exercise obese women and control group was determined using Student’s *t*-test. *Statistically significant difference.

Chronic physical exercise significantly decreased Amyg SUVmax (3.35 ± 0.9 to 2.7 ± 0.62, *p* < 0.001, **Figures 3A, B, D**) and AmygA (0.76 ± 0.17 to 0.61 ± 0.1, *p* < 0.001, **Figures 3A, B, F**). In contrast, Temporal SUVmean was not significantly changed (4.44 ± 0.75 to 4.44 ± 0.59, *p* = 0.952, **Figure 3E**). Physical exercise also reduced surrogate markers of systemic inflammation such as spleen SUVmax, BM SUVmax, and hsCRP (**Figure 2**). The effect of physical exercise on other clinical parameters is presented on **Supplementary Table 1**.

### Comparison between post-exercise obese women and control participants

AmygA was significantly highest in baseline, intermediate in post-exercise, and lowest in control group (**Figures 3A, B, C, F**). Although, exercise reduced body adiposity and systemic inflammation, post-exercise obese women still showed significant higher BMI, waist circumference, hsCRP, spleen SUVmax, and BM SUVmax than the control participants (**Figures 2A–G**). Comparison of other clinical parameters between post-exercise obese women and control participants is presented on **Supplementary Table 2**.

### Association between AmygA and systemic inflammation in obese women at baseline

AmygA exhibited significant correlation with hsCRP and BM SUVmax in obese women at baseline, whereas it showed no significant correlation with systemic inflammation surrogate markers in control participants (**Table 2**). As shown in **Table 3**, AmygA presented significant association with higher body adiposity, lower high-density lipoprotein cholesterol (HDL-C), higher BM SUVmax, and higher hsCRP. In further multivariate analysis, AmygA showed significant association with higher hsCRP. Thus, based on these findings, we concluded that AmygA was significantly associated with systemic inflammation in obese women.

**Table 2:** Correlation analysis between AmygA and surrogate markers for systemic inflammation.

Status	Parameter	AmygA	
		*r*	*p*
Baseline	hsCRP	0.688	< 0.001*
	Spleen SUVmax	0.378	0.076
	BM SUVmax	0.49	0.018*
Post-exercise	hsCRP	0.204	0.351
	Spleen SUVmax	0.366	0.086
	BM SUVmax	0.192	0.38
Control	hsCRP	-0.212	0.369
	Spleen SUVmax	0.144	0.633
	BM SUVmax	-0.293	0.21

AmygA, amygdala activity; hsCRP, high-sensitivity C-reactive protein; SUVmax, maximum standardized uptake value; BM, bone marrow.

*Statistically significant difference.

**Table 3:** Univariate- and multivariate analysis with AmygA as a dependent variable at baseline obese women .

	Univariate		Multivariate	
Variable	Coefficients (95% CI)	*p*	Coefficients (95% CI)	*p*
Age	0.002 (-0.008 – 0.011)	0.739		
BMI (kg/m^2^)	0.041 (0.011 – 0.071)	0.01*	-0.001 (-0.054 – 0.053)	0.979
Waist circumference (cm)	0.014 (0.002 – 0.027)	0.025*	0.001 (-0.017 – 0.018)	0.948
Hip circumference (cm)	0.017 (0.004 – 0.03)	0.013*	0.012 (-0.007 – 0.032)	0.186
AST (IU/L)	0 (-0.02 – 0.02)	0.98		
ALT (IU/L)	0.006 (-0.013 – 0.026)	0.515		
Triglyceride (mg/dL)	0.001 (-0.001 – 0.003)	0.376		
Total cholesterol (mg/dL)	-0.001 (-0.003 – 0.002)	0.611		
HDL-C (mg/dL)	-0.006 (-0.013 – 0)	0.045*	-0.005 (-0.01 – 0.001)	0.116
LDL-C (mg/dL)	-6.572E-005 (-0.003 – 0.003)	0.961		
Glucose (mg/dL)	-0.001 (-0.01 – 0.008)	0.856		
SBP (mmHg)	0.003 (-0.002 – 0.007)	0.192		
DBP (mmHg)	0.004 (-0.002 – 0.011)	0.199		
Spleen SUVmax	0.227 (-0.028 – 0.482)	0.079		
BM SUVmax	0.295 (0.031 – 0.558)	0.03*	0.009 (-0.347 – 0.365)	0.957
hsCRP (mg/L)	0.027 (0.008 – 0.046)	0.007*	0.023 (0.004 – 0.042)	0.021*

BMI, body mass index; AST, aspartate transaminase; ALT, alanine aminotransferase; HDL-C, high-density lipoprotein cholesterol; LDL-C, low-density lipoprotein cholesterol; SBP, systolic blood pressure; DBP, diastolic blood pressure; SUVmax, maximum standardized uptake value; BM, bone marrow; hsCRP, high-sensitivity C-reactive protein; CI, confidence interval.

*Statistically significant difference.

### Association between AmygA and systemic inflammation at post-exercise

We next monitored changes in the association between AmygA and systemic inflammation by chronic physical exercise. As shown in **Table 2**, the correlation between AmygA and systemic inflammation was no longer significant at post-exercise. In addition, AmygA became not associated with systemic inflammation after completion of chronic physical exercise in obese women (**Table 4**).

**Table 4:** Univariate- and multivariate analysis with AmygA as a dependent variable at post-exercise obese women.

	Univariate		Multivariate	
Variable	Coefficients (95% CI)	*p*	Coefficients (95% CI)	*p*
Age	-0.002 (-0.008 – 0.004)	0.44		
BMI (kg/m^2^)	0.01 (-0.008 – 0.028)	0.27		
Waist circumference (cm)	0.004 (-0.003 – 0.012)	0.245		
Hip circumference (cm)	0.001 (-0.009 – 0.011)	0.857		
AST (IU/L)	0.005 (-0.004 – 0.014)	0.263		
ALT (IU/L)	0.008 (-0.002 – 0.019)	0.122		
Triglyceride (mg/dL)	0 (-0.001 – 0.001)	0.536		
Total cholesterol (mg/dL)	0.001 (-0.001 – 0.002)	0.412		
HDL-C (mg/dL)	-0.004 (-0.009 – 0.001)	0.133		
LDL-C (mg/dL)	0.001 (0 – 0.002)	0.24		
Glucose (mg/dL)	0.004 (0 – 0.009)	0.063		
SBP (mmHg)	0.001 (-0.004 – 0.005)	0.81		
DBP (mmHg)	0 (-0.006 – 0.006)	0.899		
Spleen SUVmax	0.194 (-0.001 – 0.39)	0.051		
BM SUVmax	0.104 (-0.104 – 0.311)	0.311		
hsCRP (mg/L)	0.013 (-0.02 – 0.047)	0.42		

BMI, body mass index; AST, aspartate transaminase; ALT, alanine aminotransferase; HDL-C, high-density lipoprotein cholesterol; LDL-C, low-density lipoprotein cholesterol; SBP, systolic blood pressure; DBP, diastolic blood pressure; SUVmax, maximum standardized uptake value; BM, bone marrow; hsCRP, high-sensitivity C-reactive protein; CI, confidence interval.

*Statistically significant difference.

### Association between changes in the levels of AmygA and systemic inflammation between baseline and post-exercise

As shown in **Table 5**, changes in the levels of AmygA were significantly associated with changes in the levels of BM SUVmax and hsCRP by both univariate and multivariate analyses. Thus, the impact of exercise on reducing AmygA was significantly associated with reducing systemic inflammation in obese women.

**Table 5:** Univariate- and multivariate analysis for changes in the levels of AmygA with changes in the levels of other variables on obese women.

	Univariate		Multivariate	
Variable	Coefficients (95% CI)	*p*	Coefficients (95% CI)	*p*
BMI (kg/m^2^)	0.005 (-0.058 – 0.069)	0.867		
Waist circumference (cm)	0 (-0.014 – 0.015)	0.97		
Hip circumference (cm)	0.017 (-0.018 – 0.052)	0.325		
AST (IU/L)	-0.006 (-0.017 – 0.005)	0.269		
ALT (IU/L)	-0.01 (-0.025 – 0.004)	0.147		
Triglyceride (mg/dL)	0.001 (-0.001 – 0.002)	0.289		
Total cholesterol (mg/dL)	0 (-0.003 – 0.003)	0.906		
HDL-C (mg/dL)	-0.002 (-0.013 – 0.01)	0.779		
LDL-C (mg/dL)	-0.001 (-0.004 – 0.001)	0.259		
Glucose (mg/dL)	-0.003 (-0.011 – 0.005)	0.422		
SBP (mmHg)	0.002 (-0.003 – 0.008)	0.421		
DBP (mmHg)	0.002 (-0.008 – 0.012)	0.634		
Spleen SUVmax	0.276 (-0.065 – 0.617)	0.107		
BM SUVmax	0.312 (0.055 – 0.569)	0.02*	0.25 (0.003 – 0.498)	0.048*
hsCRP (mg/L)	0.022 (0.003 – 0.04)	0.022*	0.017 (0 – 0.035)	0.05*

BMI, body mass index; AST, aspartate transaminase; ALT, alanine aminotransferase; HDL-C, high-density lipoprotein cholesterol; LDL-C, low-density lipoprotein cholesterol; SBP, systolic blood pressure; DBP, diastolic blood pressure; SUVmax, maximum standardized uptake value; BM, bone marrow; hsCRP, high-sensitivity C-reactive protein; CI, confidence interval.

*Statistically significant difference.

## Discussion

In the present study, for the first time in human beings, we prospectively investigated the anti-stress effect of chronic physical exercise on the stress-associated metabolic activity of amygdala as assessed by ^18^F-FDG PET/CT in obese women. The present study clearly revealed that AmygA, the metabolic activity of amygdala, was elevated in obese women and was alleviated by a 3-month of physical exercise. Furthermore, physical exercise also abolished its association with systemic inflammation in obese women.

Accumulating evidence have reported that increased neural activity of amygdala can promote the development and maintenance of obesity under conditions of stress in both animal and human studies (20–22). Using genetically modified animals, Ip et al. (20) have found that increased expression of neuropeptide-Y in amygdala under stress condition, increases food consumption and decreases energy expenditure thereby accelerating obesity. In contrast, some previous animal and human studies have reported that stress facilitates the development of obesity independently of food consumption (23–26). Thus, these data suggest that elevated food intake may not be a sole contributor to stress-associated obesity.

Recently, Ishai et al. (22) report that increased neural activity of amygdala is associated with increased leukopoietic activity in bone marrow thereby upregulating systemic inflammation, which contributes to obesity development. This finding is robustly supported by previous human imaging studies by using ^18^F-FDG PET/CT that AmygA is closely associated with BM SUVmax and hsCRP (9–11), which are also consistent with our results. Thus, systemic inflammation could be a strong mediator of the association between stress-associated neurobiological activity and obesity.

The emotional response to psychological stress has been well known to activate the projections from amygdala to brainstem thereby stimulating hypothalamic-pituitary-adrenal (HPA) axis and sympathetic nervous system, which lead to elevate the levels of circulating glucocorticoids and accelerate inflammatory cell output in the bone marrow (27–30). Exercise training reduces the levels of circulating glucocorticoids and attenuates the reactivity of sympathetic nervous system to psychological stress (14, 31). Supporting these previous findings, the current study demonstrated that chronic physical exercise reduced both AmygA and surrogate markers of systemic inflammation.

AmygA was decreased with physical exercise down to about 20% of baseline, whereas post-exercise women still showed significant higher levels of AmygA and systemic inflammation than non-obese control participants. However, interestingly, the significant association between AmygA and systemic inflammation in obese women was disappeared by chronic physical exercise. Following that, AmygA was not associated with the systemic inflammation in non-obese control participants. Thus, although the detailed underlying mechanism remains unclear, our findings could support the beneficial anti-inflammatory effect of chronic physical exercise in obesity and could further illuminate a potential mechanism that emphasizes anti-inflammatory effect of chronic exercise through affecting stress-associated amygdala metabolic activity in obese women.

A growing body of evidence has emerged on the therapeutic potential of stress reduction on CVD (32). Stress reduction has demonstrated carotid plaque stabilization in older populations and reduced adverse CVD events from a randomized controlled trial (32, 33). Furthermore, based on these findings, the American Heart Association announced a statement that stress reduction has a potential benefit in CVD and encouraged further research (34). Considering these findings, assessment of stress-related neurobiological activity for monitoring treatment response and risk stratification could be crucial for management of stress on CVD. Given that amygdala is a main component of stress response, we believe that AmygA assessed by ^18^F-FDG PET/CT could be used as a surrogate marker of stress-related amygdala metabolic activity thereby measuring stress levels in clinical field.

Our study has several limitations. First, despite being a prospective design, this study was performed with small number of participants, which have a possibility to induce selection bias. Further prospective study with large populations is warranted to confirm our findings. Second, due to limited image resolution of current ^18^F-FDG PET/CT, we could not evaluate the other brain regions related to stress-neural circuit such as hippocampus. Third, we were unable to measure the direct markers of sympathetic nervous system and HPA axis activity. Fourth, we used one fixed exercise training program in this study. Variable exercise programs, modulating type, intensity, and period of exercise, could have different effect on AmygA. Furthermore, we recruited obese women between 2008 and 2009, and applied exercise program based on the AHA/ACSM guideline which was published in 2007. However, recently, updated guideline has been published since 2018 (35). Further study with updated exercise guideline is needed to confirm our findings. Fifth, we could not control all the lifestyle factors such as dietary habits and sleeping time which might potentially affect the AmygA. Sixth, current study was conducted on obese women. Further studies with balanced gender distribution are needed to evaluate gender-specific differences of AmygA in response to exercise. Finally, as shown in **Table 1**, 34.8% of obese women reported that they consumed alcohol. However, we could not evaluate the frequency and amount of alcohol use. Chronic alcohol consumption is known to affect amygdala (36, 37). Thus, alcohol consumption could be a confounding factor for interpreting AmygA. Nevertheless, our findings counterbalanced these limitations by using a non-invasive functional imaging modality to measure the metabolic activity of amygdala, to investigate the health-protective effect of exercise on stress-associated brain neural activity in obese women.

Taken together, we provide strong evidence that the 3-month of physical exercise reduced stress-associated amygdala metabolic activity, which was defined as AmygA and measured by ^18^F-FDG PET/CT and broke its association with systemic inflammation in obese women. Despite preliminary data, these findings could support the putative health protective mechanism underlying between exercise and CVD *via* stress reduction. In addition, our study further highlights the promising role of AmygA as a surrogate marker of stress-related amygdala metabolic activity for evaluating anti-stress effect of therapeutic interventions targeted to stress reduction.

## Data availability statement

The original contributions presented in the study are included in the article/**Supplementary Material**. Further inquiries can be directed to the corresponding author.

## Ethics statement

The studies involving human participants were reviewed and approved by the Ethical Committee and Institutional Review Board of Korea University Hospital. The patients/participants provided their written informed consent to participate in this study.

## Author contributions

Conceptualization: KP and SK. Data curation: KP, CJ, and HWK. Formal analysis: KP, CJ, and HWK. Investigation: KP and CJ. Methodology: KP, CJ, and HWK. Project administration: KP and SK. Validation: KP and HWK. Visualization: KP and CJ. Writing-original draft: KP. Writing-review & editing: SK. Supervision: SK. Funding acquisition: SK. All authors contributed to the article and approved the submitted version.
