# Supplementary material for: Chronic physical exercise alleviates stress-associated amygdala metabolic activity in obese women: A prospective serial 18F-FDG PET/CT study
Source: Front Endocrinol (Lausanne). 2023 Jan 5;13:1046838. doi: 10.3389/fendo.2022.1046838 (PMC9851606; doi:10.3389/fendo.2022.1046838)
Supplement: Supplementary file 1 [file Table_1.docx]

Supplementary Table 1. The effect of exercise on other clinical parameters of obese women

| **Characteristics** | **Baseline** | **Post-exercise** | ***p*** |
| --- | --- | --- | --- |
| Hip circumference (cm) | 98.6 ± 5.1 | 95.4 ± 4.6 | < 0.001* |
| AST (IU/L) | 12.3 ± 3.9 | 11.7 ± 5.1 | 0.47 |
| ALT (IU/L) | 21 ± 3.9 | 21 ± 4.2 | 0.69 |
| Glucose (mg/dL) | 89 ± 8.3 | 88 ± 9.6 | 0.47 |
| Total cholesterol (mg/dL) | 177.4 ± 30.6 | 178.3 ± 30 | 0.89 |
| Triglyceride (mg/dL) | 105.7 ± 47.3 | 112.1 ± 46.6 | 0.26 |
| HDL-C (mg/dL) | 49.9 ± 11.3 | 49.9 ± 9.2 | 0.71 |
| LDL-C (mg/dL) | 106.3 ± 29.5 | 101.3 ± 37.9 | 0.77 |

All data were expressed as mean ± standard deviation. AST, aspartate transaminase; ALT, alanine aminotransferase; HDL-C, high-density lipoprotein cholesterol; LDL-C, low-density lipoprotein cholesterol. *P*-values of hip circumference, AST, glucose, total cholesterol, and LDL-C were determined using paired *t*-test. *P*-values of ALT, triglyceride, and HDL-C were determined using Wilcoxon signed-rank test.

*Statistically significant difference.

Supplementary Table 2. Comparison of other clinical parameters between post-exercise obese women and control participants

| Parameters | Post-exercise (n = 23) | Control (n = 20) | *p* |
| --- | --- | --- | --- |
| Age (years) | 46 ± 8.0 | 48 ± 3.4 | 0.83 |
| Smoking (current), n (%) | 0 (0) | 1 (5) | 0.465 |
| Alcohol drinking, n (%) | 8 (34.8) | 7 (35) | 0.988 |
| Menopause, n (%) | 12 (52.2) | 9 (45) | 0.639 |
| Hypertension (stage I), n (%) | 4 (17.4) | 3 (15) | 1 |
| Diabetes, n (%) | 0 (0) | 1 (5) | 0.465 |
| Dyslipidemia, n (%) | 7 (30.4) | 3 (15) | 0.294 |
| Medication, n (%) | 0 (0) | 0 (0) | — |
| AST (IU/L) | 11.7 ± 5.1 | 13.6 ± 4.6 | 0.229 |
| ALT (IU/L) | 21 ± 4.2 | 18.7 ± 5.1 | 0.122 |
| Triglyceride (mg/dL) | 112.1 ± 46.6 | 108.3 ± 32 | 0.899 |
| Total cholesterol (mg/dL) | 178.3 ± 30 | 168.3 ± 27.2 | 0.265 |
| HDL-C (mg/dL) | 49.9 ± 9.2 | 55 ± 9.9 | 0.09 |
| LDL-C (mg/dL) | 101.3 ± 37.9 | 107 ± 24 | 0.9 |
| Glucose (mg/dL) | 88 ± 9.6 | 91.3 ± 11.1 | 0.241 |

All data were expressed as mean ± standard deviation. AST, aspartate transaminase; ALT, alanine aminotransferase; HDL-C, high-density lipoprotein cholesterol; LDL-C, low-density lipoprotein cholesterol. *P*-values of age, AST, ALT, total cholesterol. HDL-C, and LDL-C were determined using Student’s *t*-test. *P*-values of triglyceride, and glucose were determined using Mann–Whitney *U* test. *P*-values of smoking, hypertension, diabetes, and dyslipidemia were determined using Fisher’s exact test. *P*-values of alcohol drinking, and menopause were determined using The Pearson Chi squared (χ2) test.
